# Jianpi Qutan Decoction improves hepatic lipid metabolism in atherosclerosis mice via PPARα-CPT1α pathway regulation

**DOI:** 10.1186/s41065-025-00580-8

**Published:** 2025-10-15

**Authors:** Xiaoyu Gao, Ying Hai, Dan Ma, Lisi Liu, Ying Pei, Jie Li, Wenping Wang

**Affiliations:** 1 Department of oncology, Shengjing Hospital of China Medical University, Shenyang, China; 2 Graduate school, Liaoning University of Traditional Chinese Medicine, Shenyang, China; 3 Department of encephalopathy, Affiliated Hospital of Liaoning University of Traditional Chinese Medicine, Shenyang, China; 4 College of Integrative Medicine, Liaoning University of Traditional Chinese Medicine, Shenyang, China; 5 College of Chinese Medicine, Liaoning University of Traditional Chinese Medicine, Shenyang, China

**Keywords:** Jianpi qutan decoction, Atherosclerosis, PPARα-CPT1α pathway, Hepatic lipid metabolism, Fatty acid metabolism

## Abstract

**Objective:**

To investigate Jianpi Qutan Decoction (JPQT)’s effects on hepatic lipid metabolism via the PPARα-CPT1α pathway.

**Methods:**

Eight male C57BL/6J mice served as controls; 32 ApoE^-/-^ mice were randomized into atherosclerosis (AS), atorvastatin calcium (AC), and low/medium/high-dose JPQT groups. Prior to the intervention, therapeutic targets of JPQT were analyzed using network pharmacology to provide a theoretical basis for subsequent experiments. The AS model was induced by a 12-week high-fat diet. Hepatic triglyceride (TG) and cholesterol (TC) were measured via GPO-PAP. Glucose tolerance (GTT) and insulin tolerance (ITT) were assessed. Pro-inflammatory cytokines were analyzed by ELISA/colorimetry. Fatty acid metabolism enzymes were evaluated using kits. PPARα-CPT1α pathway mRNA and protein expression were quantified via qPCR and Western blot.

**Results:**

JPQT and AC reduced aortic plaque lipid deposition. JPQT significantly lowered hepatic TG/TC, blood glucose, insulin, and inflammation. It modulated fatty acid metabolism by promoting ACC phosphorylation, suppressing FAS and FFA while elevating FAβO, with dose-dependent efficacy. Additionally, JPQT upregulated PPARα, CPT1α, and ACOX1 mRNA and protein expression in the liver.

**Conclusion:**

JPQT may improves lipid metabolism and reduces AS progression by activating the PPARα-CPT1α pathway, with higher doses yielding stronger effects.

**Supplementary Information:**

The online version contains supplementary material available at 10.1186/s41065-025-00580-8.

## Introduction

Atherosclerosis (AS) is a chronic disease characterized by excessive lipid deposition within the arterial intima, leading to narrowing of the arterial lumen. The pathogenesis involves several theories, including lipid infiltration, inflammatory response, oxidative stress, and endothelial damage, with lipid infiltration and inflammation playing particularly significant roles [1, 2]. The liver serves as the primary site for lipid metabolism. When cholesterol intake exceeds the liver’s capacity to metabolize it, excess cholesterol accumulates in the liver, inducing hepatic inflammation. Additionally, this surplus cholesterol enters the bloodstream and deposits along the arterial walls, may contributing to plaque formation [3, 4].

Traditional Chinese Medicine (TCM) has been shown to regulate blood lipids, reduce inflammatory responses, inhibit plaque formation, and alleviate angina pectoris in coronary heart disease [5, 6]. Consequently, TCM may provide novel insights into the prevention and treatment of atherosclerosis (AS) and related conditions, meriting further investigation. Abnormal lipid metabolism is a key pathological factor and major risk for the development of typical progressive AS lesions [7]. The liver, being a critical organ for lipid metabolism, when disrupted, can lead to hyperlipidemia and exacerbate risk factors such as inflammation, oxidative stress, insulin resistance, and foam cell formation [8]. These factors contribute to the onset of cardiovascular diseases, including AS. Therefore, modulating the liver’s role in maintaining lipid homeostasis is crucial for intervening in the progression of AS.

Research has found that lipid metabolism disorders and hepatic stress-induced inflammatory responses caused by long-term high-fat diets are key factors in the development of AS [9]. Abnormal lipid metabolism, characterized by increased levels of serum triglycerides (TG), total cholesterol (TC), and low-density lipoprotein cholesterol (LDL-C), further exacerbates the onset and progression of AS [9, 10]. Peroxisome Proliferator-Activated Receptor Alpha (PPARα) is a nuclear receptor that modulates gene expression by binding to specific DNA sequences, primarily influencing the β-oxidation of fatty acids. Studies have shown that when PPARα is activated, it can increase the expression of multiple genes involved in fatty acid catabolism, including Carnitine Palmitoyltransferase 1Alpha (CPT1α) and Acyl-CoA Oxidase 1 (ACOX1) [11, 12]. Furthermore, experimental findings indicate that lutein supplementation enhances the mRNA and protein levels of PPARα and its associated downstream genes such as CPT1α and ACOX1 in the liver, which helps improve high-fat diet-induced atherosclerosis in mice and alleviates lipid metabolism disorders and oxidative stress caused by this condition [12]. This suggests the potential value of PPARα in preventing or slowing the progression of atherosclerosis. CPT1α, located on the outer mitochondrial membrane, catalyzes the first step in the β-oxidation reaction, converting long-chain fatty acids into their active acyl-carnitine form, thus serving as one of the key rate-limiting enzymes for the entire β-oxidation pathway. Research indicates that long-term intake of α-lipoic acid (ALA) significantly elevates CPT1α mRNA expression in mice, although this treatment ultimately leads to severe hepatic steatosis [13]. This seemingly paradoxical phenomenon can be mechanistically explained by the combined effects of metabolic dysregulation and oxidative stress-induced injury. From a metabolic perspective, while ALA treatment significantly upregulates the expression of β-oxidation-related genes (CPT1α and PPARα) in the livers of C57BL/6J mice, it concurrently induces systemic elevation of serum cholesterol and triglyceride levels, along with increased expression of lipid uptake/synthesis-regulating nuclear receptors (LXRα and RXRα). This “output-input” metabolic imbalance ultimately leads to net hepatic lipid accumulation [13]. Regarding oxidative stress, although ALA has demonstrated antioxidant properties and ameliorative effects on steatosis in other models, chronic high-dose administration in healthy mice paradoxically exhibits pro-oxidant effects. This phenomenon may result from the accumulation of reactive oxygen species (ROS) and lipid peroxidation byproducts generated during ALA metabolism. These changes ultimately trigger hepatocyte damage through a “second hit” mechanism, promoting the progression from simple steatosis to steatohepatitis [13].

Jianpi Qutan Decoction (JPQT), which originates from the traditional Chinese medicine decoctions Si Junzi Tang and Gualou Xiebai Banxia Tang, is a prescription used at the Affiliated Hospital of Liaoning University of Traditional Chinese Medicine for the treatment of stable angina pectoris. A multicenter, blinded, block-randomized, parallel-controlled clinical trial [14] confirmed that when JPQT is combined with conventional basic treatments, it significantly enhances the effectiveness in treating stable angina, effectively reducing angina symptoms and improving patients’ quality of life. Notably, another study on a related decoction, Gualou Xiebai Banxia Decoction, also demonstrated improvements in angina symptoms and electrocardiogram (ECG) results, although the overall evidence quality was assessed as generally low, highlighting the need for more rigorous trials to confirm these findings [15]. While clinical findings suggest benefits of JPQT in cardiovascular conditions, the molecular mechanisms, particularly those involving hepatic lipid regulation, remain underexplored. Here, we explored whether JPQT may enhances hepatic lipid metabolism via PPARα/CPT1α pathway modulation, thereby improving AS.

## Materials and methods

### Network pharmacology analysis

Information on chemical compounds was retrieved from the HERB database, a high-throughput experiment- and reference-guided platform for Traditional Chinese medicine (TCM), covering all seven medicinal herbs analyzed in this study (http://herb.ac.cn/Contact/). The target genes associated with TCM ingredients, herbs, and formulas were identified based on the chemical fingerprint similarity between TCM components and known drugs. Known atherosclerosis-related targets were gathered by screening the GeneCards database. Cytoscape (version 3.10.0) was utilized to construct a network illustrating the interaction between herbs and atherosclerosis-related targets of JPQT. To identify the biological processes (BP) of potential JPQT targets involved in atherosclerosis, gene ontology (GO) enrichment analysis was conducted. Additionally, Kyoto Encyclopedia of Genes and Genomes (KEGG) pathway enrichment analysis was performed to provide a comprehensive understanding of the mechanisms underlying JPQT’s effects on atherosclerosis. To evaluate binding affinities and interaction modes between candidate compounds and their targets, AutodockVina 1.2.2, an in silico protein-ligand docking software, was employed. The molecular structure of ENMD-2076 was obtained from PubChem Compound (https://pubchem.ncbi.nlm.nih.gov/), while the 3D coordinates of PPARα (PDB ID: 2NPA) were downloaded from the Protein Data Bank (PDB) (https://www.rcsb.org/). For docking simulations, all protein and ligand files were converted into PDBQT format, excluding water molecules and adding polar hydrogen atoms. The grid box was centered to encompass each protein’s domain, allowing for unrestricted molecular movement during docking. Molecular docking studies were carried out using Autodock Vina 1.2.2 (http://autodock.scripps.edu/).

### Animal grouping, model establishment, and drug administration

A total of eight SPF-grade C57BL/6J mouse and forty ApoE^-/-^ mouse, all male and 6 weeks old, were purchased from SPF Biotechnology (China, Beijing). The qualification numbers for the SPF-grade C57BL/6J mice (control group) and ApoE^-/-^ mice were RB3636-228335 and RB3650-228264, respectively. All mice were housed under specific pathogen-free conditions. The animal experiments were conducted in accordance with the Guidelines for the Care and Use of Laboratory Animals and approved by the Institutional Animal Care and Use Committee of China Medical University (Shenyang, China; Approval No. CMUXN2023094).

The basic maintenance feed for the mice was purchased from Changsheng Biotechnology (China, Liaoning), with the production license number SCXK-(Liao)2015-0003. The high-fat diet for the mice was obtained (ReadyDietech, China, Shenzhen), with the certificate number 23102709. The composition of the high-fat diet included 40% kcal fat, 1.25% cholesterol, 0.5% sodium cholate, and other ingredients. The fat used was cocoa butter, chosen for its high content of saturated fatty acids and unique cocoa aroma, which enhances palatability for the animals. ApoE^-/-^ mice spontaneously develop hypercholesterolemia and pre-atherosclerotic lesions in the aortic root and aortic arch. A high-fat/high-cholesterol diet accelerates this process. In this study, all mice were adapted to the basic feed for 1 week. The C57BL/6J mice served as the control group and continued to receive the standard diet. The ApoE^-/-^ mice were fed a high-fat diet for 12 weeks to establish the atherosclerosis model. After 12 weeks, the high-fat-fed ApoE^-/-^ mouse were randomly divided into four groups: atherosclerosis model (AS) group, atorvastatin calcium (3.03 mg/kg) group, Low dose JPQT group (JPQT-L, 4.48 g/kg), Middle dose JPQT group (JPQT-M, 8.96 g/kg) and high dose JPQT group (JPQT-H, 17.9 g/kg). Each experimental subgroup consisted of eight mouse. Mouse in each group were housed separately, with free access to water. They were treated via gavage for 8 weeks. The JPQT Decoction (consisting of Codonopsis pilosula, Astragalus membranaceus, Atractylodes macrocephala, Poria cocos, Trichosanthes kirilowii, Pinellia ternata, and Glycyrrhiza uralensis at a ratio of 10:10:10:10:10:6:3, with batch numbers 751230602, 2307081, 2307232, 2307171, 2305291, 2205148, and 2303271, respectively) was obtained from the Affiliated Hospital of Liaoning University of Traditional Chinese Medicine. The dose of JPQT for mice was calculated based on the “Conversion Table of Doses per Kilogram of Body Weight between Animals and Humans,” where the mouse dose is 9.1 times the human dose. The medium dose of JPQT in mice was selected as the equivalent clinical dose for adults (JPQT clinical dose: 59 g/day for a 60 kg adult, corresponding to 8.95 g/kg in mice) [16]. The high and low doses of JPQT were 2 times and 0.5 times the equivalent dose, respectively. Atorvastatin calcium tablets (H20051408,Pfizer, USA, NYC), approval number H20051408) were chosen as the positive drug due to their efficacy in improving lipid profiles, controlling atherosclerosis progression, and inhibiting foam cell formation. As previously mentioned, ApoE^-/-^ mice not exposed to a high-fat diet can still develop hypercholesterolemia and pre-atherosclerotic lesions in the proximal aorta, making them unsuitable as baseline controls. Therefore, 6-week-old C57BL/6J mice fed a standard diet were used as the baseline control for these experiments.

### Serum and tissue sample collection

At the end of the 8th week of treatment, the mice were fasted for 12 h (with free access to water) after the last administration. Blood was collected via eye puncture. The blood samples were left to stand at room temperature for 2 h, followed by centrifugation at 4℃, 12,000 × g for 15 min. The supernatant was then stored at -80℃. The mice were euthanized by cervical dislocation, and the thoracic cavity was opened to expose the heart. The aorta was perfused with phosphate-buffered saline (PBS) (G4250,Servicebio, China, Wuhan) and then dissected. Different segments of the aorta were fixed in 4% paraformaldehyde (G1101,Servicebio, China, Wuhan) or embedded in OCT compound (G6059,Servicebio, China, Wuhan) and frozen for storage at -80℃. Both gross aortic tissue and frozen sections were prepared. The above steps refer to Greenfield, 2017 and Rodrigues, 2022 [17, 18].

### Pathological morphology

Mice liver tissues were fixed in 4% paraformaldehyde for 4 h, dehydrated in ethanol solutions, and embedded in paraffin to prepare 4 μm sections. The sections were then stained with Oil Red O (Catalog No. G1015, Servicebio, Wuhan, China) and evaluated for hepatic lipid accumulation under a microscope. The aortic root and liver samples were fixed in 4% paraformaldehyde for over 24 h, followed by dehydration and embedding. Paraffin sections of 4 μm thickness were prepared and stained with hematoxylin-eosin (HE) (Catalog No. G1005, Servicebio, Wuhan, China). The sections were then mounted and imaged for documentation.

### Lipid profile analysis

Weigh approximately 0.1 g of liver tissue, add 1 mL of isopropanol, and homogenize in an ice bath. Centrifuge at 10,000 × g and 4 °C for 10 min, then collect the supernatant and place it on ice for subsequent analysis. Then, the supernatant was aspirated, Samples were added according to the kit’s instructions. Liver and serum lipid concentrations, Liver including total cholesterol (TC) (BC1985,Solarbio, China, Beijing), triglycerides (TG) (BC0625,Solarbio, China, Beijing), serum including total cholesterol (TC) (BC1985,Solarbio, China, Beijing), triglycerides (TG) (BTK009, Bioswamp, China, Wuhan) high-density lipoprotein cholesterol (HDL-C) (BTK011, Bioswamp, China, Wuhan), and low-density lipoprotein cholesterol (LDL-C) (BTK012, Bioswamp, China, Wuhan), were measured using the GPO-PAP dual-reagent microplate method.

### Glucose tolerance test (GTT) and insulin tolerance test (ITT)

Glucose tolerance test (GTT): All groups were fasted for 12 hours with no access to food or water, and the body weight of each mouse was measured. A 20% glucose solution was diluted with physiological saline to prepare an injection concentration of 7.5 μL/g. Before the test, the blood glucose level at 0 min was recorded. After intraperitoneal injection of glucose, blood glucose levels were measured at 30, 60, 90, and 120 min, respectively. Blood glucose detection was performed by disinfecting scissors with alcohol, cutting off approximately 1 mm of the terminal tissue of the mouse's tail, gently squeezing the tail to cause bleeding, inserting a GA-3 type blood glucose test strip (Sinocare Bio-sensing Technology Co., Ltd., China) into the blood glucose meter, aligning the test strip with the bleeding site of the mouse's tail to automatically absorb about 6 μL of blood sample, and obtaining the blood glucose value after 10 seconds. Insulin tolerance test (ITT): All groups were fasted for 6 hours with no access to food or water, and the body weight of each mouse was measured. A 10% insulin solution was diluted with physiological saline to prepare an injection concentration of 7.5 μL/g. Before the test, the blood glucose level at 0 min was recorded. After intraperitoneal injection of insulin, blood glucose levels were measured at 30, 60, 90, and 120 min, respectively. Blood glucose detection was performed using the same method as GTT. 

### ELISA and colorimetric assays

Before testing, leave the blood sample at 4 °C overnight to allow serum separation. Centrifuge at 4 °C, 2500–3500 × g for 20 min, then carefully collect the supernatant. Store at -80 °C. ELISA and colorimetric assays were performed according to the manufacturer’s instructions to measure the levels of pro-inflammatory cytokines, including interleukin-6 (IL-6) (MU30044,Bioswamp, China, Wuhan), tumor necrosis factor-α (TNF-α) (MU30030,Bioswamp, China, Wuhan), and interleukin-1β (IL-1β) (MU30369, Bioswamp, China, Wuhan), as well as the levels of oxidized low-density lipoprotein (ox-LDL) (MU30430, Bioswamp, China, Wuhan).

### Measurement of fatty acid metabolism-related enzyme activities

Take 1 g of liver tissue, add 9 mL of PBS, and homogenize in an ice bath. After homogenization, centrifuge the homogenate at 10,000 × g and 4 °C for 10 min, then collect the supernatant. The levels of free fatty acids (FFA) were determined using the Amplex Red Free Fatty Acid Assay Kit (MU30224, Bioswamp, China, Wuhan) The concentration of FFA in the samples was calculated based on fluorescence readings. Acetyl-CoA carboxylase (ACC) activity was assessed using a commercially available ACC Activity Assay Kit (ml602241, mlbio, China, Shanghai). The enzyme activity level was indirectly reflected by measuring the reaction products catalyzed by ACC in tissue samples. Fatty Acid Synthase (FAS) Enzyme-Linked Immunosorbent Assay (MU30628, Bioswamp, China, Wuhan). Fatty acid β-oxidation enzyme activity was evaluated using a WST-1-based commercial kit (ml331248, mlbio, China, Shanghai), which effectively reflects changes in the intracellular fatty acid oxidation rate. All experimental procedures were strictly performed in accordance with the manufacturer’s instructions.

### Western blot analysis

Liver tissues were homogenized and lysed with RIPA lysis buffer (medium strength) (WB9203,Bioswamp, China, Wuhan), and the proteins were extracted. The proteins were separated by SDS-PAGE (PW9226,Bioswamp, China, Wuhan) and transferred to PVDF (PW9230, Bioswamp, China, Wuhan) membranes. The membranes were blocked with 5% (w/v) skim milk for 2 h, washed with TBST (G0004,Servicebio, China, Beijing), and incubated with primary antibodies overnight at 4℃. The membranes were then washed with TBST and incubated with fluorescent secondary antibodies for 2 h.HRP Goat Anti-Rabbit IgG (H + L) (SAB48169,Bioswamp, China, Wuhan; at a dilution of 1:5000) After washing with TBST, the membranes were treated with ECL (PW9209,Bioswamp, China, Wuhan) reagent and imaged using a chemiluminescent gel imaging system. The relative expression of each protein was calculated as the gray value of the target protein divided by the gray value of β-actin (GB11001,Sercivebio, China, Wuhan; at a dilution of 1:3000). Western blot analysis was performed to detect the expression of SREBP1 (AF6283, Affinity Biosciences, China, Jiangsu; at a dilution of 1:1000), SREBP2 (PAB47973, Bioswamp, China, Wuhan; at a dilution of 1:1000), ACC (21923-1-AP, Proteintech, China, Wuhan; at a dilution of 1:1000), p-ACC (29119-1-AP, Proteintech, China, Wuhan; at a dilution of 1:1000), PPARα (PAB58903, Bioswamp, China, Wuhan; at a dilution of 1:1000), CPT1α (PAB33949, Bioswamp, China, Wuhan; at a dilution of 1:1000), ACOX1 (DF12046, Affinity, China, Jiangsu; at a dilution of 1:1000) in mouse liver tissues.

### Real-time PCR

Total RNA was extracted from mouse liver tissues using Trizol. The RNA was precipitated with chloroform, isopropanol, and 75% ethanol, and dissolved in DEPC-treated water. The RNA concentration and purity were measured. Genomic DNA was removed by treating 800 ng of total RNA with 2 µL of gDNA Eraser and 4 µL of 5× gDNA Eraser Buffer in a 20 µL reaction at 42℃ for 2 min, followed by storage at 4℃. cDNA was synthesized from 20 µL of the genomic DNA-removed RNA using 2 µL of RT Primer Mix, 2 µL of PrimeScript^®^ RT Enzyme Mix I, and 8 µL of 5× PrimeScript^®^ Buffer (for Real Time) in a 40 µL reaction at 37℃ for 15 min, followed by 85℃ for 5 s and storage at 4℃ (RR047A, TaKaRa, Japan, Osaka). MicroRNA cDNA was synthesized from 6 µL of total RNA using 1 µL of TransScript miRNA RT Enzyme Mix and 10 µL of 2× TS miRNA Reaction Mix in a 20 µL reaction at 37℃ for 1 h, followed by 85℃ for 5 s to inactivate the RT enzyme (RR047A, TaKaRa, Japan, Osaka).

Real-time PCR was performed using SYBR^®^ Premix Ex Taq™ II (2×) (RR420A, TaKaRa, Japan, Osaka) for mRNA detection and Universal miRNA qPCR SuperMix for microRNA detection. The PCR conditions were as follows: 95℃ for 30 s, followed by 45 cycles of 95℃ for 10 s, 60℃ for 30 s, and 95℃ for 15 s, 60℃ for 1 min. The primer sequences are listed in Table 1. The relative gene expression levels were calculated using the 2 ^− ΔΔCt^ method [19].

**Table 1:** Primer sequences

Gene symbol	Primers sequence	Product length (bp)
PPARα	Forward: AACATCGAGTGTCGAATATGTGG Reverse: CCGAATAGTTCGCCGAAAGAA	99 bp
CPT1α	Forward: TGGCATCATCACTGGTGTGTT Reverse: GTCTAGGGTCCGATTGATCTTTG	133 bp
ACOX1	Forward: CCGCCACCTTCAATCCAGAG Reverse: CAAGTTCTCGATTTCTCGACGG	86 bp
GAPDH	Forward: TGGCCTTCCGTGTTCCTAC Reverse: GAGTTGCTGTTGAAGTCGCA	178 bp

### Statistical analysis

All result data in this experiment were analyzed using IBM SPSS Statistics 22 statistical analysis software. *P* > 0.05 indicates no significant differences between groups; *P* ≤ 0.05 indicates significant differences; *P* ≤ 0.01 and *P*≤ 0.001 indicate highly significant differences, with smaller *P* values indicating greater significance. Graphs of the results were drawn using GraphPad Prism 7 software, and all result data were expressed as mean ± standard deviation (mean ± s).

## Results

### Network pharmacology predicts potential Pharmacological mechanisms for JPQT treatment of atherosclerosis

**Fig. 1:**
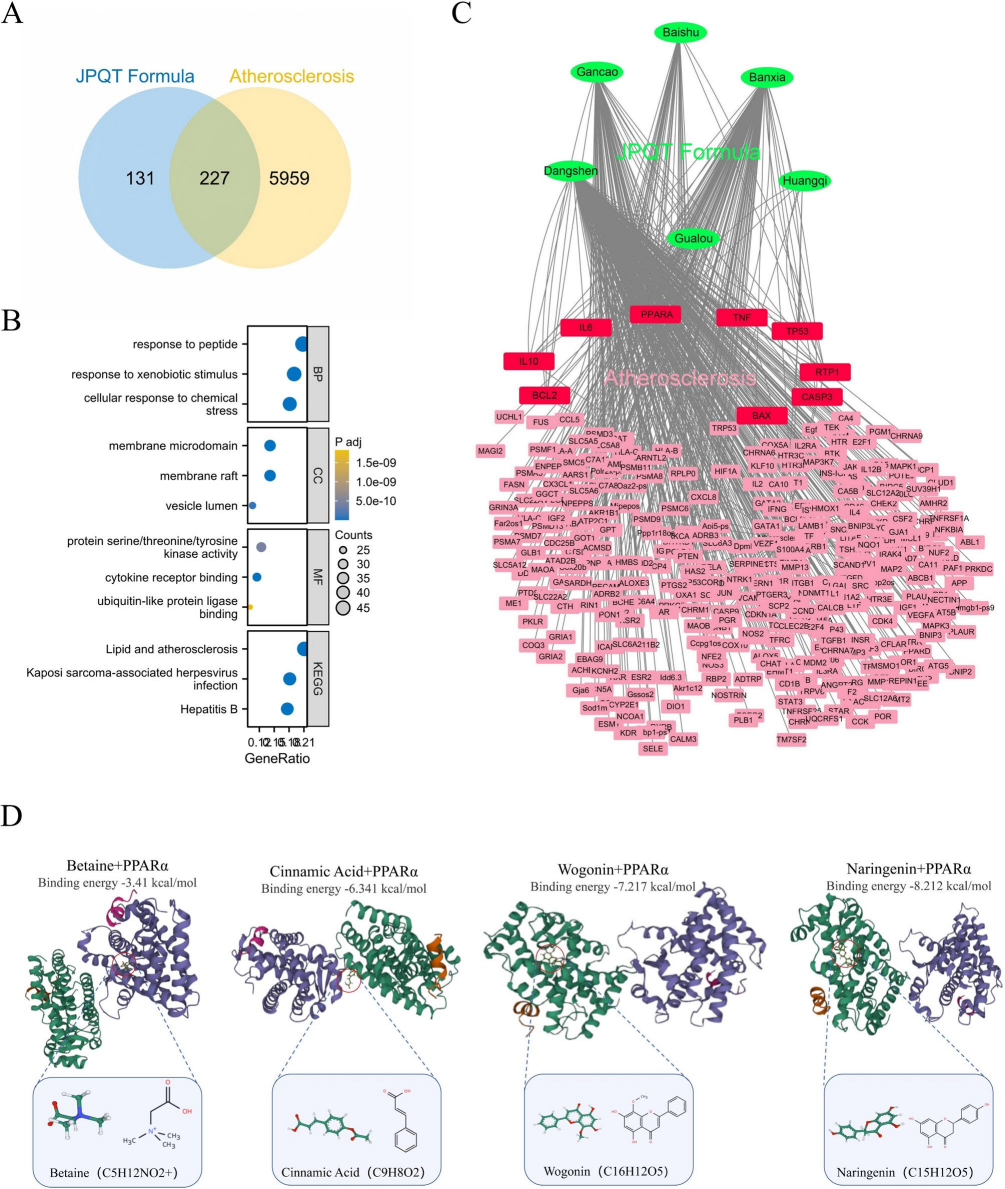
Network pharmacological prediction for JPQT treatment of atherosclerosis. **A:** Venn diagram of the corresponding targets of the effective components of JPQT and atherosclerosis genes. **B:** GO and KEGG pathway enrichment analysis. **C:** herbs-disease-target networks of JPQT against atherosclerosis. The Green oval nodes represent herbs of JPQT, the Red rectangle nodes represent atherosclerosis genes (≥ 3 effective components targeting the same gene together), and the pink rectangle nodes represent atherosclerosis genes (≤ 2 effective components targeting the same gene together). **D:** Molecular docking reveals that the four compounds of JPQT target PPARα

Using the HERB database, we predicted 358 target genes for JPQT. By employing a Venn diagram to screen for common target genes between JPQT and atherosclerosis, we identified 227 shared target genes (Fig. 1A). Subsequently, these 227 shared target genes were subjected to GO and KEGG enrichment analyses. The top enriched items in the biological process (BP) category were “response to peptide” and “response to xenobiotic stimulus.” In the cellular component (CC) category, the top enriched terms were “membrane raft” and “membrane microdomain.” For the molecular function (MF) category, the most significantly enriched items were “cytokine receptor binding” and “protein serine/threonine/tyrosine kinase activity.” Finally, in the KEGG pathway analysis, the top enriched term was “lipid and atherosclerosis” (Fig. 1B).

To facilitate the visualization and further interpretation of the target prediction results, we constructed a compound-target (C-T) network. PPARα, a key molecule involved not only in lipid metabolism regulation but also in modulating inflammatory responses affecting atherosclerosis, was highlighted in this network. The C-T network revealed that PPARα is targeted by four JPQT compounds: Betaine, Naringenin, Cinnamic Acid, and Wogonin (Fig. 1C). Betaine originates from Astragalus membranaceus, while Naringenin is derived from both Astragalus membranaceus and Codonopsis pilosula. Cinnamic Acid is sourced from Astragalus membranaceus, Codonopsis pilosula, and Pinellia ternata. Wogonin is found in Astragalus membranaceus and Glycyrrhiza uralensis.

To evaluate the binding affinity of these four JPQT compounds for PPARα, we conducted molecular docking analysis. The binding poses and interactions of these compounds with PPARα were assessed using AutoDock Vina v.1.2.2, and the binding energy for each interaction was calculated (Fig. 1D). The results demonstrated that all four JPQT compounds bound to PPARα through visible hydrogen bonds and strong electrostatic interactions. Additionally, the hydrophobic pockets of PPARα were successfully occupied by these compounds. Regarding KDR, the binding energies of Betaine, Naringenin, Cinnamic Acid, and Wogonin were − 3.41, -8.212, -6.341, and − 7.217 kcal/mol, respectively, indicating highly stable binding interactions.

### The pathological changes of aorta and liver were observed by H&E staining

**Fig. 2:**
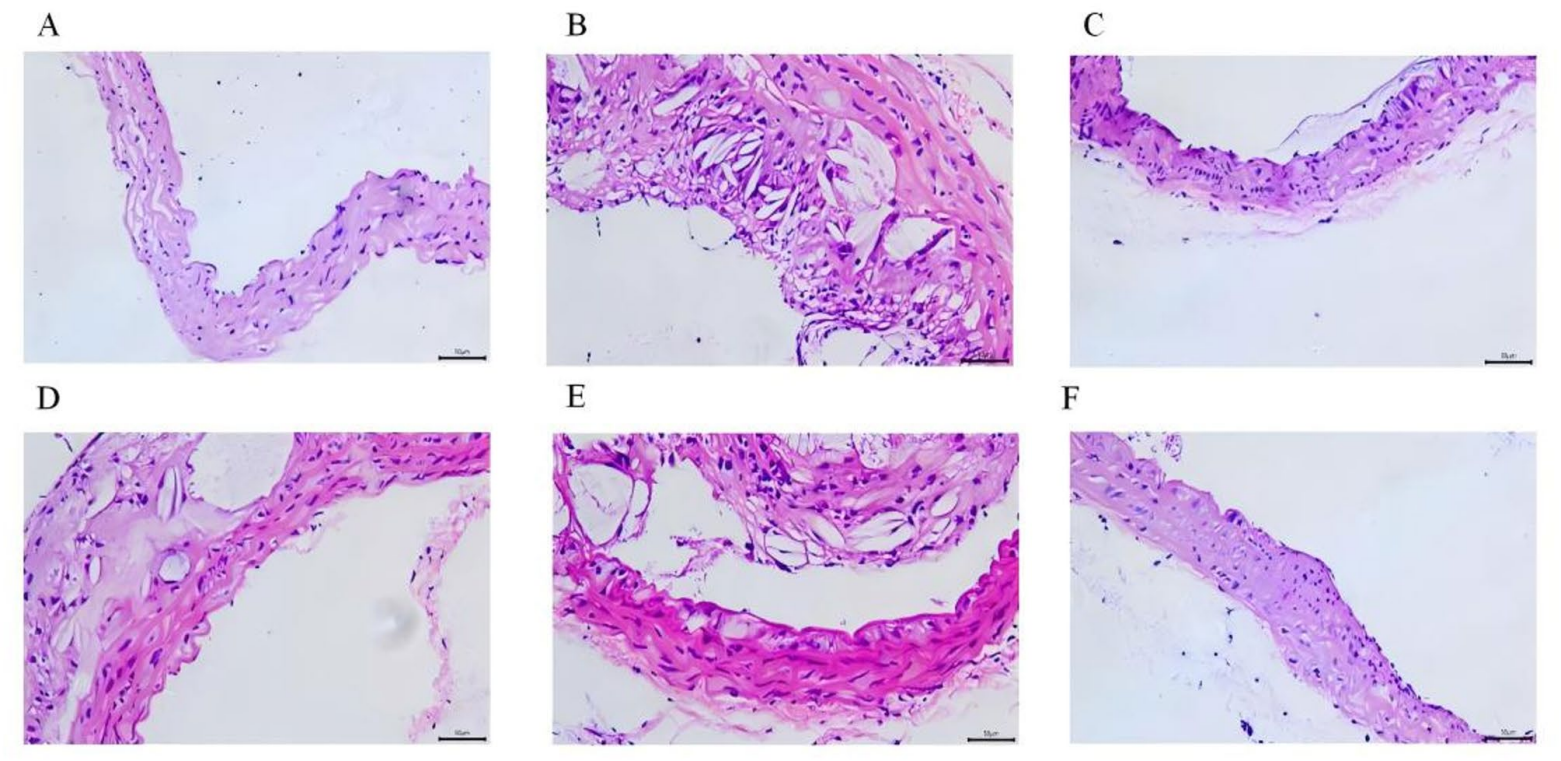
Aorta was observed with HE staining. **A-F**: CON: control group; AS: atherosclerosis model group; AC: atorvastatin calcium group; JPQT-L (Low dose JPQT group); JPQT-M (Middle dose JPQT group) and JPQT-H (high dose JPQT group). Bar = 50 μm

**Fig. 3:**
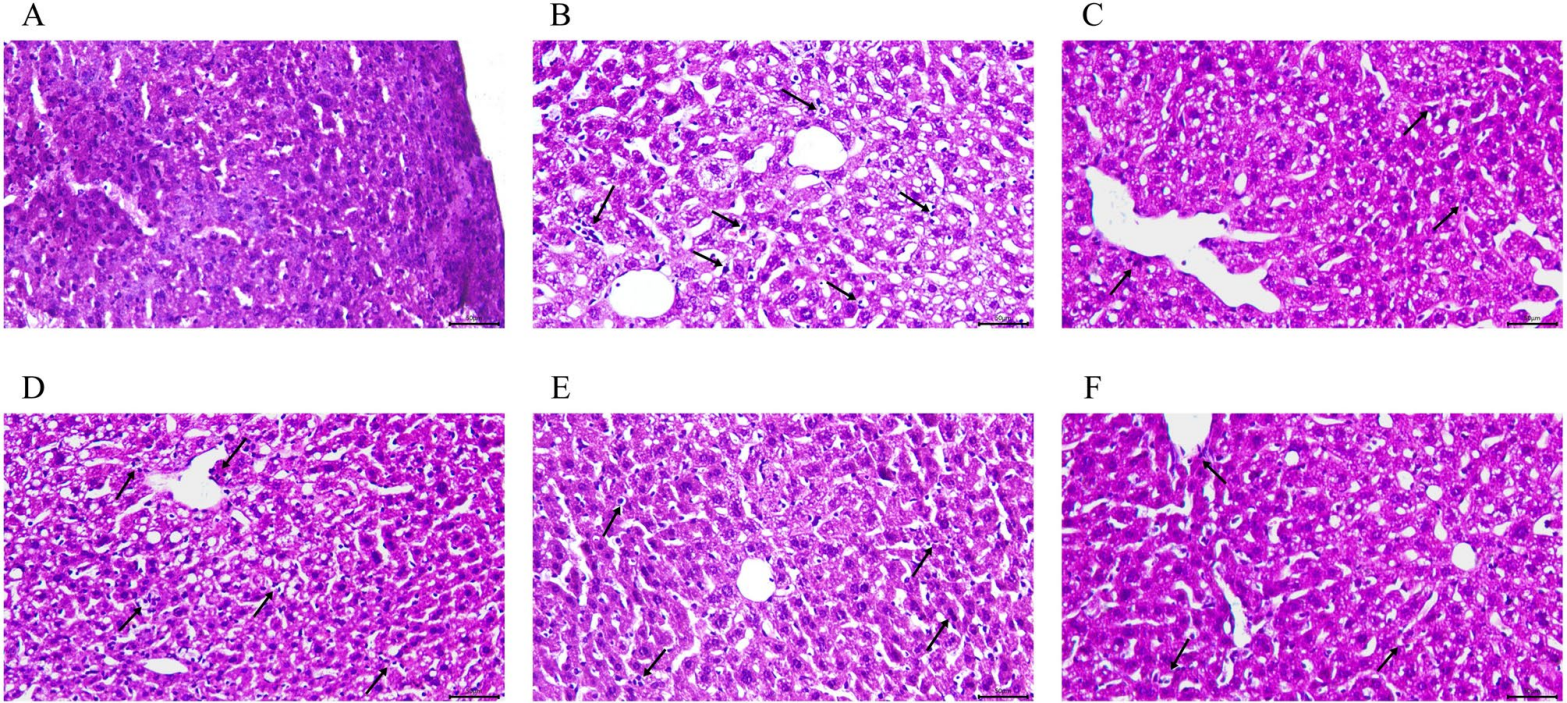
Liver was observed by HE staining. **A-F**: CON: control group; AS: atherosclerosis model group; AC: atorvastatin calcium group; JPQT-L (Low dose JPQT group); JPQT-M (Middle dose JPQT group) and JPQT-H (high dose JPQT group). The arrow points to the inflammatory cells. Bar = 50 μm

Aortic plaque area in AS group mice was markedly larger than in the CON group. After 12 weeks of drug treatment, JPQT-H and AC groups showed a significant decrease in aortic plaque area (Fig. 2). In the CON group, liver histology displayed well-defined hepatic lobule structures, distinct confluent area boundaries, and hepatocytes arranged radially around the central vein, with intact hepatic sinusoids (Fig. 3). Conversely, AS group mice exhibited hepatocyte swelling and disrupted hepatic cord organization. Compared to the AS group, the AC and JPQT-H groups demonstrated restored hepatocyte morphology, orderly hepatic cords, and an absence of lipid droplet vacuoles in central venous cells. Additionally, to assess the safety of JPQT, we performed the same HE staining on other organs of the mice, including the heart, spleen, lung, and kidney. The results showed that JPQT at any dose did not cause histological changes in these areas (Supplementary Material).

### The pathological changes of liver were observed by oil red O staining

**Fig. 4:**
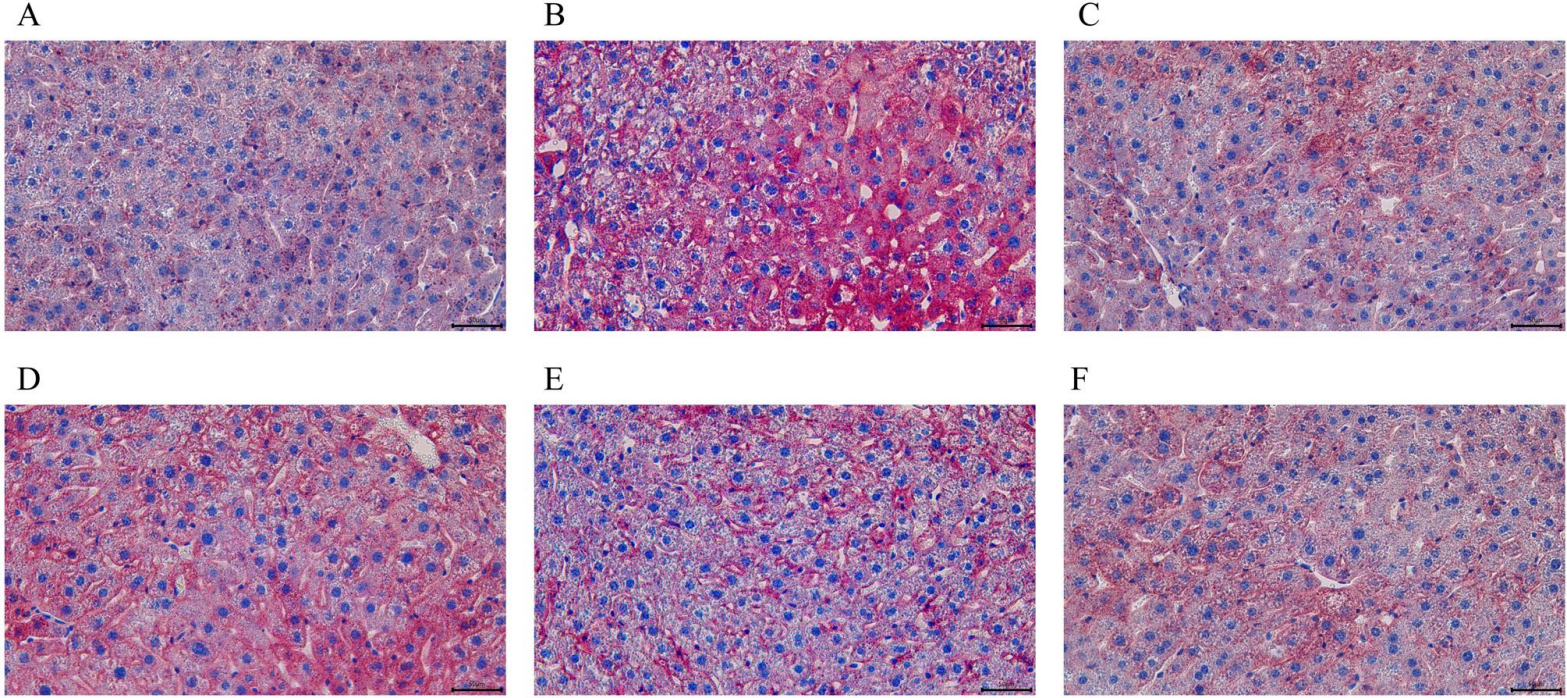
Observation of lipid deposition in mouse liver using Oil Red O staining. **A:** CON: Control group; **B:** AS: Atherosclerosis model group; **C:** AC: Atorvastatin calcium group; **D:** JPQT-L (JPQT low-dose group); **E:** JPQT-M (JPQT medium-dose group); **F:** JPQT-H (JPQT high-dose group). Scale bar = 50 μm

As shown in Fig. 4, the Oil Red O staining results indicated that the liver of the control group mice exhibited normal cellular arrangement and minimal lipid deposition. In contrast, the intervention group mice showed significant hepatic lipid accumulation, characterized by a marked increase in red lipid droplets within the liver, disordered cellular arrangement, and an increased number of fat vacuoles, suggesting abnormal hepatic lipid metabolism and steatosis. Compared with the model group, the AC group and the JPQT group showed a significant reduction in red-stained lipid droplets in the liver, more orderly hepatocyte arrangement, a decreased number of fat vacuoles, and alleviated hepatic lipid deposition, with JPQT demonstrating a dose-dependent effect.

### The detection of serum lipid levels and blood glucose

**Fig. 5:**
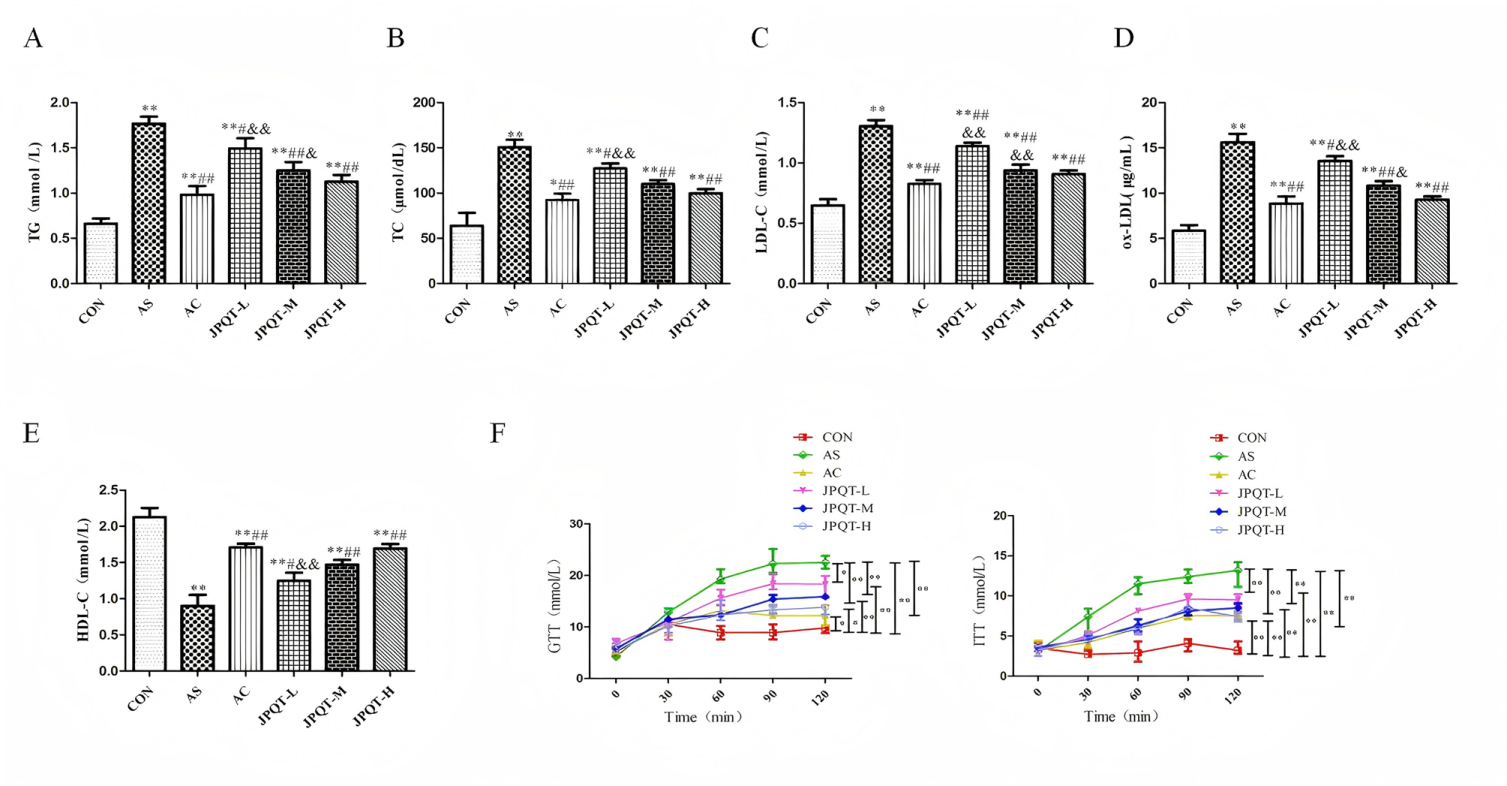
GPO-PAP double reagent microplate method and kit detection of serum lipid and blood glucose. (A) TG, (B) TC, (C) LDL-C,（D）ox-LDL, (E) HDL-C and (F) blood glucose and insulin. CON: control group; AS: atherosclerosis model group; AC: atorvastatin calcium group; JPQT-L (Low dose JPQT group); JPQT-M（Middle dose JPQT group）and JPQT-H (high dose JPQT group). **P < 0.01 and *P < 0.05 compared with CON group; ##*P* < 0.01and #*P* < 0.05 compared with AS group; &&*P*< 0.01 and &*P*< 0.05 compared with AC group. n=8.

**Fig. 6:**
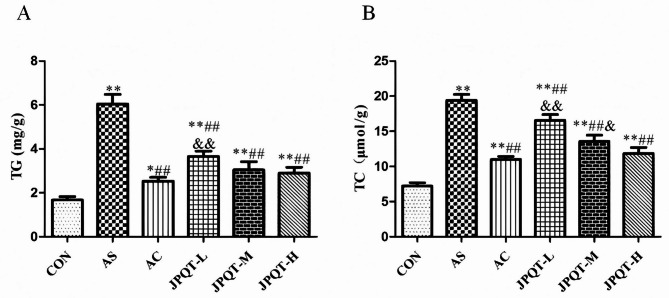
GPO-PAP double reagent microplate method was used to detect liver biochemical indexes.(A) TG, (B) TC. CON: control group; AS: atherosclerosis model group; AC: atorvastatin calcium group; JPQT-L (Low dose JPQT group); JPQT-M（Middle dose JPQT group）and JPQT-H (high dose JPQT group). **P < 0.01 and *P < 0.05 compared with CON group; ##*P* < 0.01and #*P* < 0.05 compared with AS group; &&*P*< 0.01 and &*P*< 0.05 compared with AC group. n=8.

The results of serum lipids and blood glucose detection were shown in Fig.5 , which showed that the levels of TG, TC, LDL-C as well as ox-LDL were significantly reduced (*P* < 0.05) and the levels of HDL-C were significantly increased (*P < 0.01*) in the AC and JPQT groups as compared to the AS group (Fig. 5A-E), and GTT, ITT detection showed that (Fig. 5F), the levels of blood glucose and insulin were significantly reduced (*P* < 0.01; *P*< 0.05) in the AC and JPQT groups as compared to the AS group. In addition, the effect of JPQT was enhanced with increasing concentration. The liver levels of TG (*P* < 0.01), TC (*P* < 0.01) were markedly lower in the AC and JPQT groups when compared to the AS group (Fig. 6), And the effect of JPQT was enhanced with increasing concentration.

### The detection of inflammatory cytokine levels in serum

**Fig. 7:**
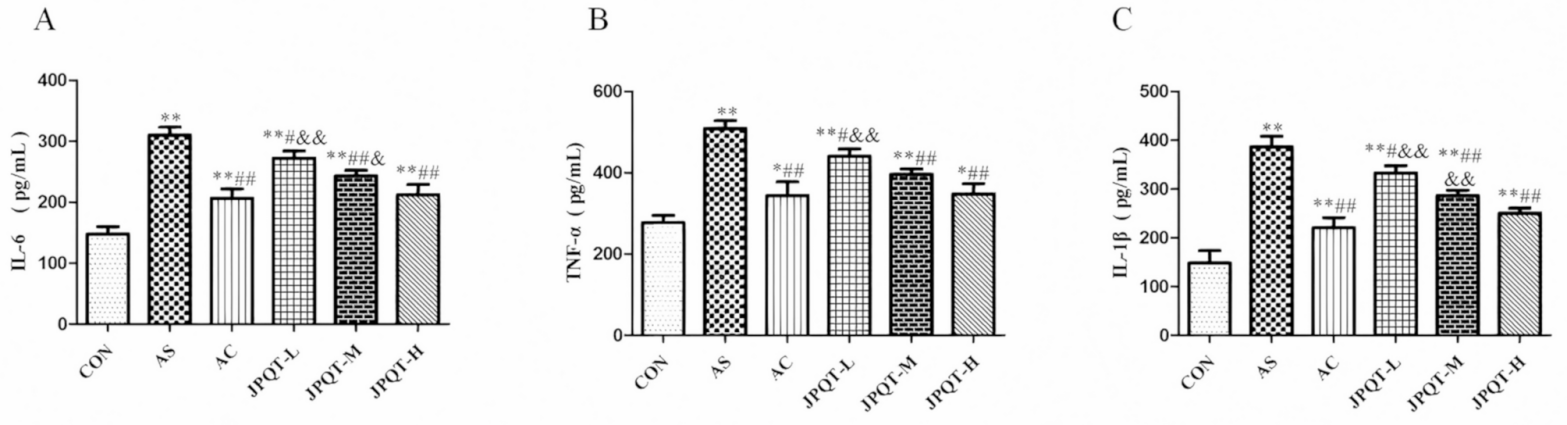
Detection of serum inflammatory factors by ELISA method. (A) IL-6, (B) TNF-α, (C) IL-1β. CON: control group; AS: atherosclerosis model group; AC: atorvastatin calcium group; JPQT-L (Low dose JPQT group); JPQT-M（Middle dose JPQT group）and JPQT-H (high dose JPQT group).**P < 0.01 and *P < 0.05 compared with CON group; ##*P* < 0.01 and #*P* < 0.05 compared with AS group; &&*P*< 0.01 and &*P*< 0.05 compared with AC group. n=8.

Compared to the CON group, serum IL-6 levels in the AS group were significantly elevated (*P* < 0.01), indicating that the modeling successfully induced an inflammatory response. Serum IL-6 levels in both the AC group and the JPQT-H group were significantly lower than those in the AS group (*P* < 0.01). Serum TNF-α levels in the AS group were significantly higher than those in the CON group (*P* < 0.01), further confirming the successful establishment of the AS inflammation model. Serum TNF-α levels in the AC group (*P* < 0.01), JPQT-L group (*P* < 0.05) and JPQT-H group (*P* < 0.01) were significantly lower than those in the AS group. Among these, the AC group showed the greatest reduction, followed by the JPQT-H group and the JPQT-L group, indicating a dose-dependent effect of JPQT. Serum IL-1β levels in the AS group were significantly higher than those in the CON group (*P* < 0.01), demonstrating the involvement of IL-1β in the inflammatory response. Serum IL-1β levels in the AC group (*P* < 0.01), JPQT-L group (*P* < 0.05), and JPQT-H group (*P* < 0.01) were significantly lower than those in the AS group. The AC group exhibited the greatest reduction, followed by the JPQT-H group and the JPQT-L group, further supporting the dose-dependent effect of JPQT. Both AC and JPQT effectively suppressed serum levels of IL-6, TNF-α, and IL-1β in mice (Fig. 7). The AC group demonstrated the best therapeutic efficacy, while JPQT exhibited a dose-dependent effect, with higher concentrations leading to better treatment outcomes.

### The detection of proteins related to hepatic lipid metabolism in mouse liver

**Fig. 8:**
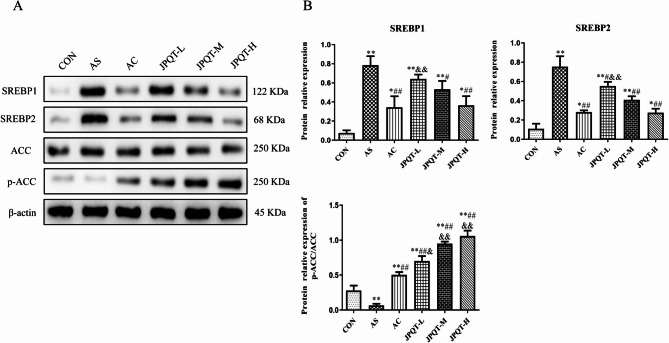
Western blot analysis protein expression levels of SREBP1, SREBP2, ACC and p-ACC in the liver. (A) Western blot image. (B) Quantitative analysis of SREBP1, SREBP2, ACC and p-ACC protein expression levels.CON: control group; AS: atherosclerosis model group; AC: atorvastatin calcium group; JPQT-L (Low dose JPQT group); JPQT-M (Middle dose JPQT group) and JPQT-H (high dose JPQT group). **P < 0.01 and *P < 0.05 compared with CON group; ##*P* < 0.01 and #*P* < 0.05 compared with AS group; &&*P*< 0.01 and &*P*< 0.05 compared with AC group. n=8.

The levels of Sterol Regulatory Element Binding Protein 1 (SREBP1), Sterol Regulatory Element Binding Protein 2 (SREBP2), Acetyl-CoA Carboxylase (ACC), and phosphorylated Acetyl-CoA Carboxylase (p - ACC) were detected by western blot. In the results (Fig. 8), for the SREBP1 index, its expression level was the highest in the AS group (*P* < 0.01), followed by JPQT - L group (*P* < 0.01), JPQT - M group (*P* < 0.01). The expression levels of the JPQT - H group were the same as those of the AC group and lower than the previous three groups (*P* < 0.01), while the CON group had the lowest expression level. The expression trend of the SREBP2 index was similar to that of SREBP1. Regarding the ACC index, there were no obvious differences among the groups. For the p - ACC index, the JPQT - H group had the highest expression level, followed by the JPQT - M group, JPQT - L group, and AC group. The expression level of the CON group was higher than that of the AS group. This implies that the expressions of SREBP1 and SREBP2 are up - regulated during the development of atherosclerosis, and JPQT may have a dose - dependent inhibitory effect on their expressions, with the high - dose group showing an inhibitory effect comparable to that of atorvastatin calcium. The lack of differences in ACC among the groups suggests that its protein expression level is not affected by JPQT. The significantly increased expression of p - ACC in the JPQT - H group indicates that JPQT may regulate lipid metabolism by promoting the phosphorylation of ACC, and the higher the dose, the more obvious the effect. This may be one of the important mechanisms by which JPQT improves lipid metabolism disorders associated with atherosclerosis.

### The detection of fatty acid metabolism-related enzyme content in mouse liver

**Fig. 9:**
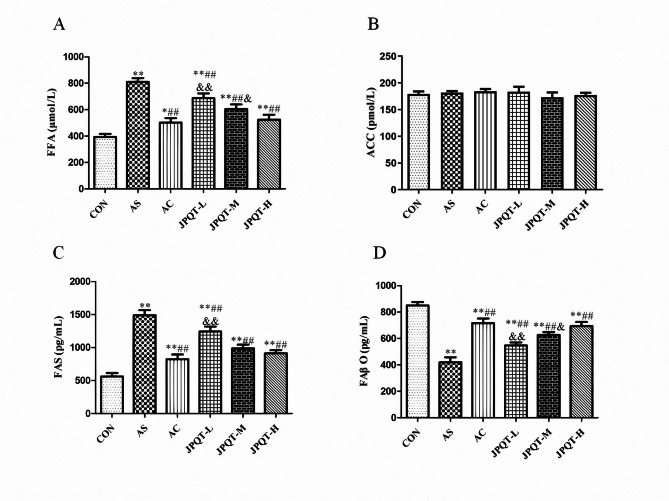
Measurement of Fatty Acid Metabolism-Related Enzyme content by ELISA method. (A)FFA, (B) ACC, (C) FAS, (D) FAβO. CON: control group; AS: atherosclerosis model group; AC: atorvastatin calcium group; JPQT-L (Low dose JPQT group); JPQT-M（Middle dose JPQT group）and JPQT-H (high dose JPQT group). **P < 0.01 and *P < 0.05 compared with CON group; ##*P* < 0.01 and #*P* < 0.05 compared with AS group; &&*P*< 0.01 and &*P*< 0.05 compared with AC group. n=8.

The content of free fatty acids (FFA), the content of acetyl-CoA carboxylase (ACC), the content of fatty acid synthetase (FAS) and the content of fatty acid β-oxidation enzymes in mouse liver were measured to evaluate the regulatory effects of drugs on hepatic fatty acid metabolism (Fig. 9). Compared to the CON group, the FFA content in the liver of the AS group was significantly increased (*P* < 0.01), indicating disordered fatty acid metabolism in the AS group. The FFA content in the AC group (*P* < 0.01), JPQT-L group (*P* < 0.01), and JPQT-H group (*P* < 0.01) were significantly lower than those in the AS group, suggesting that drug treatments effectively inhibited hepatic FFA accumulation. Among these, the AC group showed the most significant reduction, followed by the JPQT-H and JPQT-L groups, indicating a dose-dependent effect of the herbal treatment. The detection of ACC content in the liver of each group showed that there was no significant difference in ACC content among the AS group, CON group, AC group, low-dose JPQT group, and high-dose JPQT group (*P > 0.05*). This indicates that under the experimental conditions of this study, different treatment groups did not have a significant impact on the ACC content in the liver, which is consistent with the previous results. The fatty acid β-oxidation content in the liver of the AS group was significantly lower than that in the CON group (*P* < 0.01), indicating impaired fatty acid oxidation in the AS group. The FAS content in the AC group (*P* < 0.01), JPQT-L group (*P* < 0.01), and JPQT-H group (*P* < 0.01) were significantly lower than those in the AS group, suggesting that drug treatments effectively inhibited hepatic FAS accumulation. Among these, the AC group showed the most significant reduction, followed by the JPQT-H and JPQT-L groups, indicating a dose-dependent effect of the herbal treatment. The fatty acid β-oxidation enzyme content in the AC group (*P* < 0.01), JPQT-L group (*P* < 0.01), and JPQT-H group (*P* < 0.01) were significantly higher than those in the AS group, suggesting that drug treatments effectively promoted hepatic fatty acid β-oxidation. The AC group showed the greatest enhancement, followed by the JPQT-H and JPQT-L groups, indicating a dose-dependent effect of the herbal treatment. The results demonstrate that the drugs effectively regulated hepatic fatty acid metabolism by inhibiting FFA content and FAS content while promoting fatty acid β-oxidation enzyme content. The AC group exhibited the best therapeutic efficacy, and the herbal treatment showed a dose-dependent effect, with higher concentrations leading to better outcomes.

### The mRNA expression levels of PPARα, CPT1α and ACOX1 in the liver

**Fig. 10:**
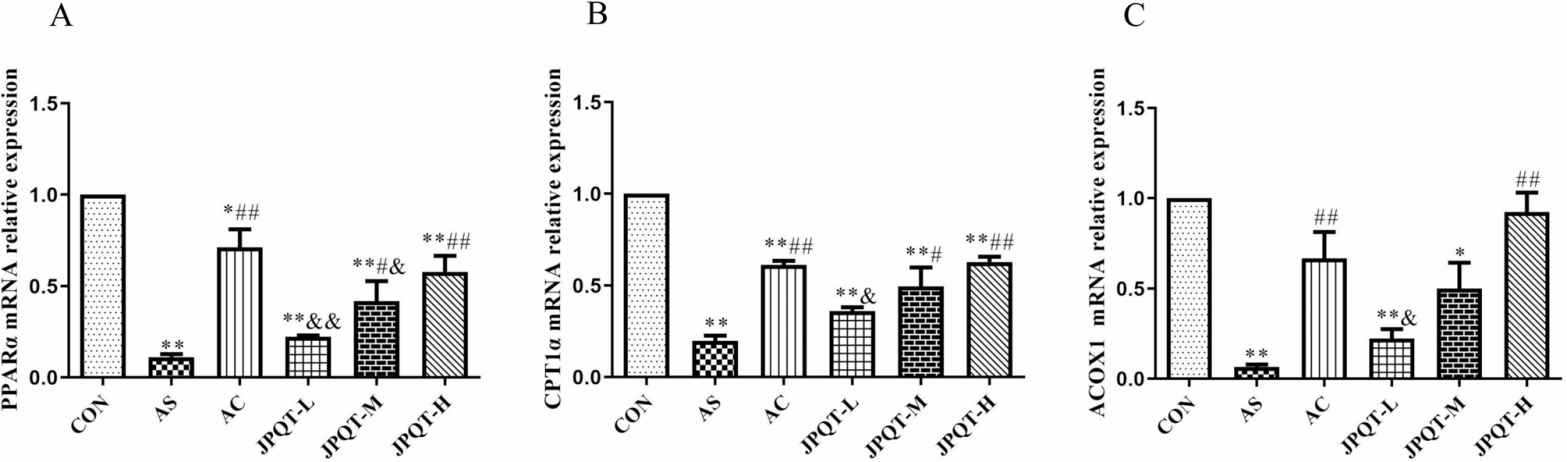
Detect mRNA expression levels in mice liver by RT-PCR. (A) PPARα, (B) CPT1α, (C) ACOX1. CON: control group; AS: atherosclerosis model group; AC: atorvastatin calcium group; JPQT-L (Low dose JPQT group); JPQT-M（Middle dose JPQT group）and JPQT-H (high dose JPQT group). **P < 0.01 and *P < 0.05 compared with CON group; ##*P* < 0.01and #*P* < 0.05 compared with AS group; &&*P*< 0.01 and &*P*< 0.05 compared with AC group. n=8.

As illustrated in Fig. 10, the hepatic mRNA expression levels of PPARα, CPT1α and ACOX1 were markedly decreased in the AS group mice compared to those in the CON group (*P* < 0.01, *P* < 0.01 and *P* < 0.01, respectively). In contrast, the hepatic mRNA expression levels of PPARα, CPT1α and ACOX1 were significantly increased in JPQT-H group when compared to the AS group (*P* < 0.01, *P* < 0.01 and *P* < 0.01, respectively).

### The protein expression levels of PPARα, CPT1α and ACOX1 in the liver

**Fig. 11:**
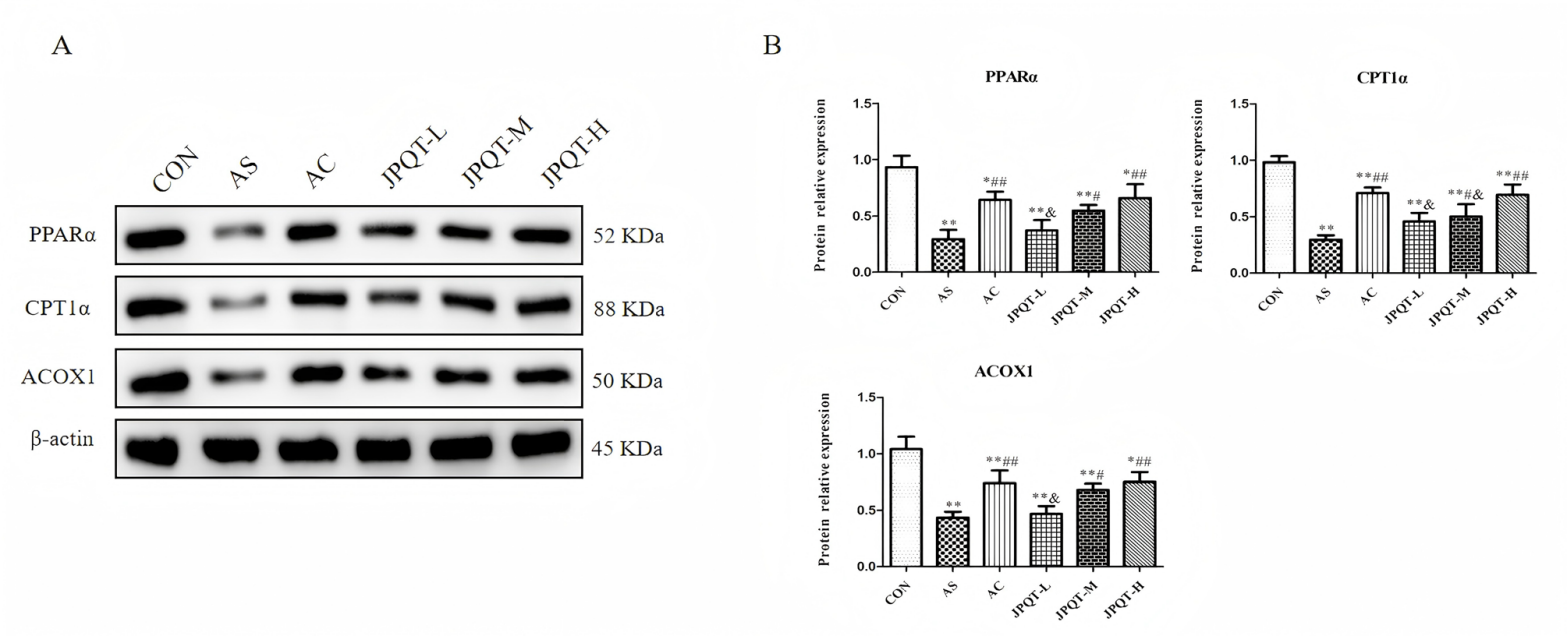
Western blot analysis protein expression levels of PPARα, CPT1α and ACOX1 in the liver. (A) Western blot image. (B) Quantitative analysis of PPARα, CPT1α, ACOX1 protein expression levels. **P < 0.01 and *P < 0.05 compared with CON group; ##*P* < 0.01 and #*P* < 0.05 compared with AS group; &&*P*< 0.01 and &*P*< 0.05 compared with AC group. n=8.

As illustrated in Fig. 11, the hepatic protein expression levels of PPARα, CPT1α, and ACOX1 in AS group were notably decreased compared to those in the CON group (*P* < 0.01, *P* < 0.01 and *P* < 0.01, respectively). Furthermore, the hepatic protein expression levels of PPARα, CPT1α, ACOX1 were significantly increased in JPQT-H group when compared to the AS group (*P* < 0.01, *P* < 0.01 and *P* < 0.01, respectively).

## Discussion

Atherosclerosis is characterized by the buildup of lipids, inflammatory cells, and fibrous components in arterial walls, leading to plaque formation and potential thrombotic events such as myocardial infarction and strokes [20]. The disease progresses through several stages, including endothelial dysfunction, oxidative stress, inflammatory response, fatty streak formation, and plaque maturation [21]. The development of atherosclerosis is multifactorial, involving both genetic and environmental influences. Key contributing factors include dyslipidemia, oxidative stress, chronic low-grade inflammation, immune dysregulation, and vascular dysfunction [22]. The pathogenesis and progression of AS are jointly regulated by inflammatory factors, lipid profile distribution, and lipid metabolism, which interact to form a complex pathological network. Inflammation, as one of the central drivers of AS, is mediated by key cytokines such as C-reactive protein (CRP), interleukin (IL)-6, and tumor necrosis factor (TNF)-α. These mediators promote vascular endothelial injury and exacerbate plaque formation and destabilization at various stages of AS [23]. Inflammatory cytokines amplify vascular wall inflammation through activation of signaling pathways such as NF-κB [24]. In this study, the inflammatory response played a critical role in AS pathogenesis, as evidenced by significantly elevated serum levels of pro-inflammatory cytokines (IL-6, TNF-α, and IL-1β) in the AS model group compared with control. These findings confirm successful induction of inflammation in the model and demonstrate the involvement of these cytokines in the pathological process of AS. Measurement of inflammatory cytokine levels provides a means to evaluate the degree of inflammation and disease progression in AS. Following treatment, both the AC and JPQT groups exhibited significant reductions in serum inflammatory cytokine levels, indicating therapeutic efficacy in suppressing inflammatory responses and ameliorating AS progression. Dyslipidemia represents another pivotal pathological factor in AS.

When cholesterol intake exceeds hepatic metabolic capacity, excess cholesterol accumulates in the liver and subsequently enters the bloodstream, depositing within arterial walls to initiate plaque formation [25]. Lipid profile distribution serves as a crucial indicator for assessing cardiovascular disease risk. Traditional biomarkers for atherosclerosis include low-density lipoprotein (LDL), high-density lipoprotein (HDL) and triglycerides (TG). Specific types of lipids, such as low-density lipoprotein (LDL) cholesterol, oxidized LDL (ox-LDL), and small dense LDL (sdLDL), play critical roles in the onset and progression of the disease [26]. Assessment of lipid profile distribution enables the prediction of AS risk and provides a basis for disease prevention and treatment. In the present study, the AS model group exhibited significantly elevated serum levels of TG, TC, LDL-C, and ox-LDL, accompanied by reduced HDL-C levels. These aberrant lipid profile alterations collectively contributed to the initiation and progression of AS. Additionally, metabolic changes, including abnormal expression of multiple genes and factors, lead to the accumulation of lipids and the activation of immune responses, further exacerbating the condition [27].

The liver serves as the primary site for lipid metabolism, and abnormalities in lipid metabolic parameters can lead to dysregulated lipid metabolism, thereby triggering metabolic disorders such as fatty liver disease. In this study, key lipid metabolic markers, including FFA, ACC, FAS, and fatty acid β-oxidation enzymes, were examined. The results revealed significantly elevated hepatic levels of FFA and FAS, along with reduced fatty acid β-oxidation enzyme activity in AS model mice, indicating disrupted hepatic fatty acid metabolism. Furthermore, alterations in lipid metabolic parameters reflect dysregulation in lipid metabolic pathways. The PPARα-CPT1α pathway plays a pivotal role in lipid metabolism, and its activation enhances fatty acid β-oxidation while reducing lipid accumulation [28]. Notably, JPQT treatment significantly upregulated both mRNA and protein expression levels of PPARα, CPT1α, and ACOX1, suggesting that JPQT may ameliorates AS progression by modulating hepatic lipid metabolism via activation of the PPARα-CPT1α pathway.

These three aspects—inflammation, lipid profile distribution, and lipid metabolism—exhibit intricate crosstalk. Inflammatory cytokines modulate lipid metabolic enzyme activity, thereby altering lipid distribution, while dyslipidemia exacerbates AS progression by inducing chronic inflammation. Concurrently, abnormal lipid profiles further amplify inflammatory responses, creating a self-perpetuating pathological cycle. Thus, elucidating the synergistic mechanisms among inflammation, lipid distribution, and metabolism not only unveils the pathological essence of AS but also provides a theoretical foundation for therapeutic strategies aimed at suppressing inflammation, optimizing lipid profiles, and restoring metabolic homeostasis. Current treatments for atherosclerosis encompass lifestyle modifications, pharmacological interventions, and surgical procedures. Lifestyle changes, such as diet and exercise, are fundamental in managing the disease. Pharmacological treatments include statins, which are the primary agents used to manage unstable plaques by reducing cholesterol levels, enhancing endothelial function, and mitigating inflammatory responses. While statins are effective in lowering cholesterol and reducing cardiovascular risk, they can cause side effects such as muscle pain, liver enzyme abnormalities, and, rarely, rhabdomyolysis [27].

Recent research has highlighted the roles of PPARα, along with its downstream targets ACOX1 and CPT1α, in the pathogenesis and potential treatment of atherosclerosis. These studies collectively underscore the importance of these genes in regulating lipid metabolism, inflammation, and vascular health. In a study conducted by Engel et al., it was observed that in patients undergoing carotid endarterectomy (CEA) and major lower extremity amputations, the expression levels of PPARα and its downstream genes, ACOX1 and CPT1α, were significantly higher in minimally diseased arterial segments compared to maximally diseased ones, especially in diabetic patients [29]. This finding suggests a possible compensatory mechanism where increased PPARα signaling may attempt to mitigate the progression of atherosclerosis in less severely affected areas. The study also found that gene expression correlated with clinical markers such as Hemoglobin A1c and serum lipid profiles, indicating a potential link between systemic metabolic status and local vascular PPARα activity. Further supporting the role of PPARα in modulating lipid metabolism, Su and Kaluzny’s work demonstrated that microRNA-378a, which is upregulated in response to a high-fructose diet, suppresses the expression of ERRγ, PPARα, CPT1α, and ACOX1, all of which are involved in fatty acid β-oxidation (FAO) [30]. Another key aspect of PPARα in atherosclerosis is its anti-inflammatory properties. Li et al. reviewed the multifaceted roles of PPARα in cardiovascular diseases, including its ability to inhibit pro-inflammatory signaling pathways and improve lipid profiles [31]. They noted that PPARα agonists have shown promise in reducing atherosclerotic plaque formation and improving endothelial function. The development of selective PPARα modulators (SPPARMα) has opened new avenues for treating dyslipidemia and atherosclerosis. Hennuyer et al. demonstrated that pemafibrate, a novel SPPARMα, not only improves lipid profiles but also enhances reverse cholesterol transport and reduces inflammation and atherosclerosis [32]. This study highlights the potential of targeted PPARα modulation as a therapeutic approach. Lastly, Zheng et al. provided a comprehensive review on the spatial and temporal roles of PPARs in atherosclerosis, emphasizing the importance of stage- and cell type-dependent precision therapy [33]. They suggested that PPARs, particularly PPARα, play a critical role in the early stages of atherosclerosis by improving endothelial function and regulating macrophage lipid metabolism. In later stages, PPARs can help reduce fibrous cap formation and stabilize plaques. These findings suggest that targeting PPARα and its downstream pathways may offer promising therapeutic strategies for the prevention and treatment of atherosclerosis.

Traditional Chinese medicine (TCM) has long recognized the importance of the spleen in maintaining overall health, particularly in relation to digestion and the metabolism of fats. The concept of strengthening the spleen and removing phlegm is based on the TCM theory that an impaired spleen function can lead to the accumulation of phlegm and dampness, contributing to metabolic disorders such as atherosclerosis and nonalcoholic fatty liver disease (NAFLD). Recent studies have highlighted the potential of natural products, including traditional Chinese herbal formulations, in the treatment of atherosclerosis and related lipid metabolism disorders. Specifically, these natural products are being explored for their ability to modulate peroxisome proliferator-activated receptors (PPARs), which play a crucial role in regulating lipid metabolism and inflammatory responses [34]. By regulating the spleen and stomach, the treatment has demonstrated significant efficacy in clinical practice. Yang J et al. investigated the molecular mechanisms underlying the protective effects of three classic TCM Decoctions (Sijunzi, Lizhong, and Fuzilizhong) on NAFLD [35]. These decoctions, which are known for their spleen-strengthening and phlegm-removing properties, were found to significantly improve liver health by reactivating PPARα signaling, reducing hepatic damage, and alleviating inflammation and fibrosis in high-fat diet-induced NAFLD in rats. The activation of PPARα, a key regulator of fatty acid oxidation, was particularly important in the context of lipid metabolism. Study had shown that natural compounds could enhance the expression of PPARα, leading to increased fatty acid β-oxidation and reduced lipid accumulation [36]. Additionally, the upregulation of ACOX1 and CPT1α, both of which were downstream targets of PPARα, further supports the beneficial effects of these compounds on lipid metabolism. In the case of Sijunzi, Lizhong, and Fuzilizhong decoctions, the reactivation of PPARα signaling was accompanied by the upregulation of ACOX1 and CPT1α, suggesting that these decoctions promote fatty acid oxidation and reduce lipid deposition. This finding provides a mechanistic basis for the observed improvements in liver health and the mitigation of NAFLD symptoms [35].

In this study, we found that JPQT effectively reduced aortic plaque area, alleviated hepatic lipid deposition, and significantly lowered liver TG, TC, LDL-C, and blood glucose levels in atherosclerosis (AS) model mice. Additionally, the mRNA and protein expression levels of PPARα, CPT1α, ACOX1, which were associated with anti-lipid accumulation, were markedly decreased in the livers of AS mice. In contrast, JPQT treatment significantly enhanced the expression of these biomarkers. Our findings suggest that JPQT mitigated AS lesions by effectively regulating lipid metabolism in the mouse liver, and its mechanism of action may related to the activation of the PPARα-CPT1α pathway.

PPARα can promote the synthesis of a series of enzymes related to fatty acid catabolism, including CPT1α [37]. As the rate-limiting enzyme for fatty acid entry into mitochondria for β-oxidation, the activity of CPT1α directly determines the fatty acid oxidation rate. Therefore, by increasing the expression level or activity of CPT1α, PPARα can significantly enhance cellular fatty acid utilization efficiency and help reduce serum triglyceride and cholesterol concentrations [38]. In the hyperglycemic state observed in obesity or type 2 diabetes, AMPK (AMP-activated protein kinase) is activated to counteract energy stress, and AMPK itself is one of the key upstream regulators of the PPARα/CPT1α axis [39]. AMPK can upregulate the expression of PPARα and its target genes such as CPT1α through phosphorylation, thereby promoting fatty acid oxidation and inhibiting glucose production in the liver and other tissues, achieving the effect of improving insulin resistance [40]. CPT1α, an enzyme located on the outer mitochondrial membrane, is responsible for transporting long-chain fatty acids into mitochondria for β-oxidation to generate energy. Consequently, when the PPARα/CPT1α signaling pathway is activated, cells tend to increase fatty acid breakdown rather than synthesis and storage. This effect is crucial for combating obesity and related metabolic diseases such as non-alcoholic fatty liver disease (NAFLD). Studies have demonstrated that activation of PPARα does not directly alter the protein content of ACC, but rather regulates its activity by promoting its phosphorylation. AMP-activated protein kinase (AMPK) is one of the key factors governing ACC. Activation of PPARα can induce phosphorylation of ACC through AMPK-mediated signaling, thereby inactivating ACC [41, 42]. This phosphorylation primarily occurs at specific serine residues of ACC (e.g., Ser-79 and Ser-1200), and phosphorylation at these sites significantly reduces the enzymatic activity of ACC [43]. Consequently, although the expression level of ACC may remain unchanged, its catalytic activity is inhibited by phosphorylation, which reduces the production of malonyl-CoA and promotes fatty acid oxidation [41]. Another study revealed that in cardiac tissues, AMPK-mediated phosphorylation of ACC not only diminishes its activity but also alters its functional mode associated with fatty acid metabolism [44].

## Conclusion

Overall, our research highlighted the potential of JPQT in the treatment of atherosclerosis and related lipid metabolism disorders through the modulation of PPARα, ACOX1 and CPT1α. Based on animal experimental results, JPQT demonstrates potential in treating atherosclerosis and related lipid metabolism disorders, and is expected to become an alternative to synthetic drugs. It should be emphasized that although this study demonstrated a dose-dependent effect of JPQT on improving lipid metabolism and attenuating atherosclerosis progression, the optimal dosage range has not been established. Furthermore, potential toxicological risks associated with JPQT overdose remain uninvestigated. Therefore, further studies are warranted to determine the safe therapeutic window and safety profile of JPQT, thereby providing more robust evidence for its clinical application. Overall, further studies are needed to fully understand the effective active substances, and the mechanisms to develop novel therapeutic strategies based on JPQT.

## Supplementary Information

Below is the link to the electronic supplementary material.

Supplementary Material 1

Supplementary Material 2

## Data Availability

All of the data generated during this study were included in this article.
