# Supplementary figures and images for: Jianpi Qutan Decoction improves hepatic lipid metabolism in atherosclerosis mice via PPARα-CPT1α pathway regulation
Source: Hereditas. 2025 Oct 15;162:209. doi: 10.1186/s41065-025-00580-8 (PMC12522855; doi:10.1186/s41065-025-00580-8)

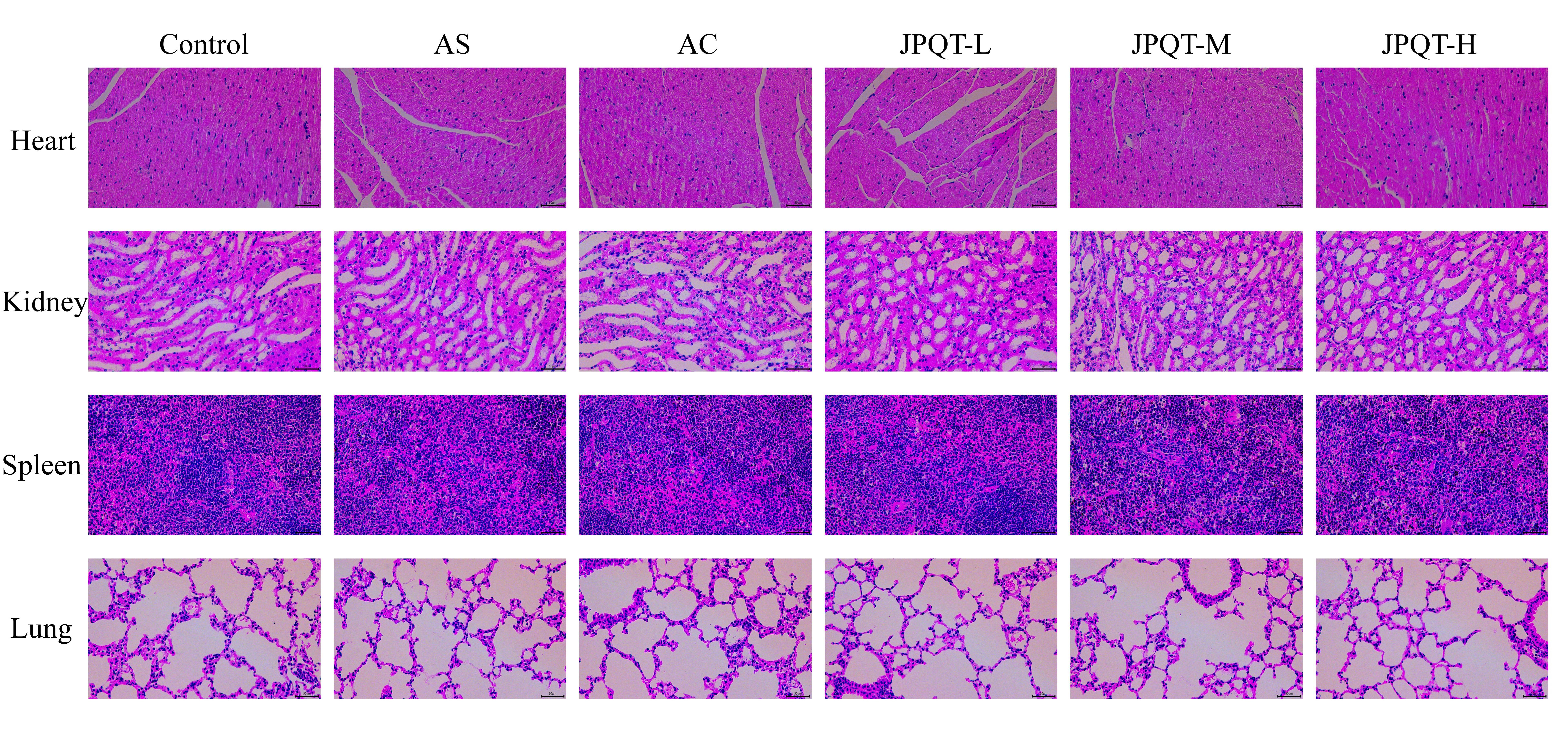

Supplement: Supplementary file 1 — Supplementary Material 1 [file 41065_2025_580_MOESM1_ESM.jpg]
