# Supplementary material for: Jianpi Qutan Decoction improves hepatic lipid metabolism in atherosclerosis mice via PPARα-CPT1α pathway regulation
Source: Hereditas. 2025 Oct 15;162:209. doi: 10.1186/s41065-025-00580-8 (PMC12522855; doi:10.1186/s41065-025-00580-8)
Supplement: Supplementary file 2 — Supplementary Material 2 [file 41065_2025_580_MOESM2_ESM.docx]

**Supplement Table 1**. Weight changes of mice in each group during treatment

|  | Control | AS | AC | JPQT-L | JPQT-M | JPQT-H |
| --- | --- | --- | --- | --- | --- | --- |
| 1 | 22.5g±0.5g | 30g±0.5g | 30g±0.3g | 30g±0.6g | 30g±0.7g | 30g±0.5g |
| 2 | 22.9g±0.3g | 31.4g±0.3g | 30.8g±0.2g | 31.2g±0.4g | 31g±0.5g | 30.8g±0.5g |
| 3 | 23.3g±0.4g | 32.7g±0.2g | 31.5g±0.4g | 32.3g±0.5g | 31.9g±0.4g | 31.5g±0.6g |
| 4 | 23.7g±0.4g | 34g±0.4g | 32.1g±0.5g | 33.5g±0.6g | 32.8g±0.5g | 32.3g±0.7g |
| 5 | 24.1g±0.4g | 35.3g±0.4g | 32.6g±0.4g | 34.5g±0.5g | 33.6g±0.5g | 33g±0.5g |
| 6 | 24.6g±0.5g | 36.6g±0.5g | 33g±0.4g | 35.4g±0.5g | 34.3g±0.6g | 33.6g±0.4g |
| 7 | 25.2g±0.5g | 37.9g±0.5g | 33.6g±0.6g | 36.4g±0.4g | 35g±0.5g | 34.2g±0.5g |
| 8 | 25.8g±0.6g | 39.2g±0.6g | 34.3g±0.5g | 37.3g±0.4g | 35.6g±0.7g | 34.8g±0.6g |

**Supplement Table 2**. Food intake of mice in each group during treatment

|  | Control | AS | AC | JPQT-L | JPQT-M | JPQT-H |
| --- | --- | --- | --- | --- | --- | --- |
| 1 | 3.2g±0.3g | 3g±0.2g | 2.8g±0.3g | 2.7g±0.3g | 2.7g±0.5g | 2.9g±0.2g |
| 2 | 3.1g±0.4g | 2.8g±0.4g | 2.9g±0.2g | 2.9g±0.4g | 2.8g±0.4g | 3g±0.5g |
| 3 | 3.2g±0.2g | 3g±0.3g | 3g±0.2g | 3.1g±0.4g | 3g±0.6g | 2.9g±0.6g |
| 4 | 3g±0.4g | 3.2g±0.4g | 3.1g±0.4g | 3.2g±0.5g | 3.1g±0.5g | 3g±0.6g |
| 5 | 3.1g±0.5g | 3.3g±0.3g | 2.9g±0.3g | 2.9g±0.4g | 2.9g±0.5g | 3g±0.3g |
| 6 | 2.9g±0.4g | 3.3g±0.3g | 3.1g±0.5g | 3.3g±0.4g | 3.1g±0.5g | 3.1g±0.5g |
| 7 | 3.2g±0.5g | 3.1g±0.4g | 2.8g±0.4g | 2.7g±0.5g | 2.8g±0.3g | 2.9g±0.3g |
| 8 | 3.1g±0.3g | 3.2g±0.3g | 3.2g±0.3g | 3.1g±0.6g | 3.2g±0.4g | 3.1g±o.4g |
